# Substantially Delayed Maturation of Growth Plate Chondrocytes in “Humanized” *PTH1R* Mice with the H223R Mutation of Jansen's Disease

**DOI:** 10.1002/jbm4.10802

**Published:** 2023-08-15

**Authors:** Monica Reyes, Damla Firat, Patrick Hanna, Mohd Khan, Michael Bruce, Maria Shvedova, Tatsuya Kobayashi, Ernestina Schipani, Thomas J. Gardella, Harald Jüppner

**Affiliations:** ^1^ Endocrine Unit Massachusetts General Hospital and Harvard Medical School Boston MA USA; ^2^ Department of Orthopedic Surgery University of Pennsylvania, Perelman Medical School Philadelphia PA USA; ^3^ Pediatric Nephrology Unit Massachusetts General Hospital and Harvard Medical School Boston MA USA

**Keywords:** ANIMAL MODELS, BIOCHEMICAL MARKERS OF BONE TURNOVER, BONE MODELING AND REMODELING, CELL/TISSUE SIGNALING – ENDOCRINE PATHWAYS, CHONDROCYTE AND CARTILAGE BIOLOGY, GENETIC ANIMAL MODELS, GROWTH PLATE, PTH/Vit D/FGF23

## Abstract

Activating parathyroid hormone (PTH)/PTH‐related Peptide (PTHrP) receptor (PTH1R) mutations causes Jansen's metaphyseal chondrodysplasia (JMC), a rare disease characterized by growth plate abnormalities, short stature, and PTH‐independent hypercalcemia. Previously generated transgenic JMC mouse models, in which the human *PTH1R* allele with the H223R mutation (*H223R‐PTH1R*) is expressed in osteoblasts via type Ia1 collagen or DMP1 promoters cause excess bone mass, while expression of the mutant allele via the type IIa1 collagen promoter results in only minor growth plate changes. Thus, neither transgenic JMC model adequately recapitulates the human disease. We therefore generated “humanized” JMC mice in which the *H223R‐PTH1R* allele was expressed via the endogenous mouse *Pth1r* promoter and, thus, in all relevant target tissues. Founders with the H223R allele typically died within 2 months without reproducing; several mosaic male founders, however, lived longer and produced F1 *H223R‐PTH1R* offspring, which were small and exhibited marked growth plate abnormalities. Serum calcium and phosphate levels of the mutant mice were not different from wild‐type littermates, but serum PTH and P1NP were reduced significantly, while CTX‐1 and CTX‐2 were slightly increased. Histological and RNAscope analyses of the mutant tibial growth plates revealed markedly expanded zones of type II collagen‐positive, proliferating/prehypertrophic chondrocytes, abundant apoptotic cells in the growth plate center and a progressive reduction of type X collagen‐positive hypertrophic chondrocytes and primary spongiosa. The “humanized” *H223R‐PTH1R* mice are likely to provide a more suitable model for defining the JMC phenotype and for assessing potential treatment options for this debilitating disease of skeletal development and mineral ion homeostasis. © 2023 The Authors. *JBMR Plus* published by Wiley Periodicals LLC on behalf of American Society for Bone and Mineral Research.

## Graphical abstract

The activating H223R mutation, the most frequent cause of Jansen's Metaphyseal Chondrodysplasia (JMC), was introduced via *in utero* Cas‐9 gene editing into “humanized” PTH1R mice. In H223R mice, the zones of proliferating collagen type II (col II)‐positive chondrocytes were progressively enlarged without normal layers of well‐organized collagen type X (col X)‐positive hypertrophic chondrocytes, thus recapitulating the growth plate phenotype in JMC patients (scale bar, 3 mm).

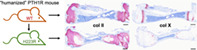

## Introduction

Jansen's metaphyseal chondrodysplasia (JMC) is an ultra‐rare autosomal‐dominant disease caused by heterozygous, activating mutations in the *PTH1R* gene that encodes the PTH/PTHrP receptor.^(^ ^1^, ^2^, ^3^ ^)^ Thus far, five different *PTH1R* mutations have been identified in JMC patients that affect one of three different amino acid residues, His223, Thr410, and Ile458, located at the intracellular portion of transmembrane helix 2, 6, and 7, respectively.^(^ ^4^, ^5^ ^)^ The JMC mutations lead to constitutive, agonist‐independent cAMP formation, as measured in cell‐based assays in vitro.^(^ ^5^, ^6^ ^)^ This agonist‐independent activity of the mutant PTH1R leads in patients to marked growth plate abnormalities, which resemble radiographically those seen in patients with severe rickets.^(^ ^7^, ^8^ ^)^ The impact on the growth plates leads to short stature and bowing of the long bones in JMC patients, who may, furthermore, exhibit micrognathia, hypertelorism, high‐arched palate, scoliosis, and premature closure of cranial sutures,^(^ ^5^ ^)^ as well delayed tooth eruption or tooth impaction that are typically caused by heterozygous inactivating PTH1R mutations.^(^ ^9^, ^10^ ^)^

The PTH/PTHrP receptor mediates the actions of two endogenous peptides, parathyroid hormone (PTH) and PTH‐related peptide (PTHrP), and can activate at least two signaling pathways, cAMP/PKA and Ca^2+^/IP3/PKC. The PTH1R is a class B G protein‐coupled receptor (GPCR) that is abundantly expressed in kidney and bone and at very high levels in the metaphyseal growth plates.^(^ ^11^ ^)^ In the growth plates, paracrine activation of the PTH1R by PTHrP slows the differentiation of chondrocytes, thereby preventing premature growth plate closure and allowing normal bone elongation.^(^ ^12^ ^)^ In bone, endocrine activation of the PTH1R by PTH directly stimulates osteoblasts and osteocytes, which in turn regulates osteoclasts indirectly through the RANK/RANKL system,^(^ ^13^ ^)^ to promote bone resorption and, thus, calcium mobilization. In distal renal tubules, the PTH1R mediates the PTH‐dependent reabsorption of calcium, while in proximal renal tubules it enhances excretion of phosphate and expression of CYP27B1, the gene encoding the 1α‐hydroxylase.^(^ ^13^, ^14^ ^)^

Laboratory abnormalities reported for JMC patients change with age and include severe PTH‐ and PTHrP‐independent hypercalcemia and hypophosphatemia.^(^ ^5^, ^15^, ^16^ ^)^ Histomorphometric changes in bone include high rates of bone turnover, increased cortical erosions and thinning, increased cancellous bone volume, and increased rates of bone formation and mineral apposition.^(^ ^15^, ^16^ ^)^ Severe skeletal changes and life‐long evidence for hypercalcemia and/or hypercalciuria are thus hallmarks encountered in most JMC patients.^(^ ^4^, ^17^, ^18^ ^)^ However, one JMC patient with the most frequent H223R mutation revealed, possibly because of mosaicism, no overt hypercalcemia or hypophosphatemia.^(^ ^18^ ^)^ Furthermore, the T410R mutation in the PTH1R causes a less severe form of the disease without obvious serum calcium and phosphate abnormalities and heights that are at the lower end of the normal range.^(^ ^19^ ^)^ All JMC patients appear to have an increased risk of developing nephrocalcinosis and possibly impaired renal function later in life.^(^ ^5^, ^15^ ^)^

There is currently no medical treatment available for patients affected by Jansen's disease. Some PTH inverse agonist (PTH‐IA) peptides, however, can reduce constitutive cAMP accumulation not only in vitro^(^ ^20^, ^21^, ^22^ ^)^ but also in vivo, as shown by studies in mice transgenically expressing the *PTH1R* with the H223R mutation (*H223R‐PTH1R*) under the control of the collagen type Ia1 promoter (C1‐HR mice). Treatment of these mice with a PTH inverse agonist reduces excess trabecular bone formation and moderately improves bone length and shape.^(^ ^23^ ^)^ A limitation of that prior study, however, is that C1‐HR mice express the *H223R‐PTH1R* mainly in bone‐forming osteoblasts and thus do not accurately recapitulate the main phenotypic hallmarks of JMC. Moreover, overexpression of the human H223R mutant protein targeted specifically to bone cells through transgenic methods could compromise the efficacy of PTH‐IA, even when given at high doses. Thus, a severe increase in bone mass is encountered in C1‐HR mice^(^ ^24^ ^)^ as well as in mice expressing *H223R‐PTH1R* under the control of the DMP1 promoter,^(^ ^25^ ^)^ which is not seen in JMC patients. Transgenic mice expressing the H223R mutant under the control of the collagen type IIa1 promoter (C2‐HR mice), and hence targeting the constitutively active PTH1R to growth plate chondrocytes, develop only mild and transient growth plate abnormalities.^(^ ^26^ ^)^ In addition, there has so far been no animal model in which a constitutively active mutant PTH1R was expressed in kidney, thereby preventing assessment of agonist‐independent cAMP formation in different portions of the renal tubules. A model with expression of the *H223R‐PTH1R* at physiological levels in all target tissues and cells responding to PTH or PTHrP is thus needed to gain more meaningful insights into the different abnormalities that are triggered by a constitutively active PTH1R in JMC patients and to enable a more suitable approach to evaluating potential treatment modalities for this disease.

We recently reported a mouse in which the exons encoding the membrane‐embedded portion and most of the amino‐terminal, extracellular domain of the endogenous mouse *Pth1r* gene were replaced by a cDNA encoding the human PTH1R.^(^ ^27^ ^)^ Hence, this “humanized” knock‐in mouse expresses the human *PTH1R* under the control of the endogenous mouse promoter and, hence, in all tissues that normally express this receptor and at the proper times. Homozygous *PTH1R* mice show normal growth and skeletal structures, as well as normal mineral ion homeostasis, and therefore offered the opportunity to generate mice expressing the PTH1R with the H223R mutation, the most common cause of Jansen's disease, under proper spatiotemporal control. The resulting JMC mice appear to recapitulate the key abnormalities that occur in the human disease, as we detected clear changes in growth plate chondrocyte maturation properties over time, as well as changes in blood markers of mineral ion homeostasis and bone turnover.

## Materials and Methods

### Generation of “humanized” PTH1R mice with the H223R mutation by i‐GONAD

The animal experiments in this study were approved by the Institutional Animal Care and Use Committee (IACUC) of Massachusetts General Hospital.

**Fig. 1:**
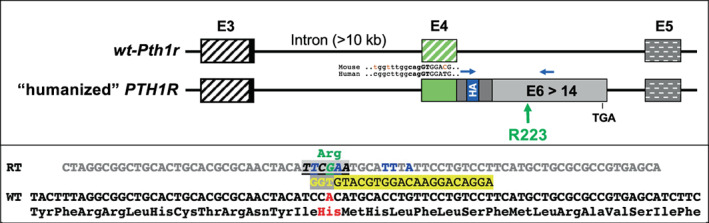
Strategy to generate *H223R‐PTH1R* mice. *Upper panel*: Portions of the WT mouse *Pth1r* (*wt‐Pth1r*) gene were replaced with the cDNA encoding the human PTH1R (*PTH1R*). The signal peptide is encoded by mouse exon 3 (E3) (formerly exon S), depicted by the black striped square, which also includes the first three amino acid residues of the mature mouse receptor protein (Tyr, Ala, and Leu; black square in E3). Starting with the segment of the PTH1R encoded by exon 4 (E4) (**GT**GGA …; green square), the remaining cDNA of the receptor was fused to the 3′ end (…gg**cag**) of the large mouse intron between exons E3 and E4. The influenza hemagglutinin epitope tag (HA‐tag; blue square) is located in the portion of the PTH1R encoded by exon 5 (E5) (gray square), followed by the cDNA encoded by human exons E6 through E14, and the location of the TGA termination codon in E14 is indicated; mouse E5 is shown by the gray squares with white broken horizontal lines. The approximate position of the introduced amino acid residue R223 (green letter/numbers) is indicated by a green arrow; positions for forward and reverse primers for amplification of the human cDNA are indicated. *Lower panel*: Portions of WT cDNA encoding the human PTH1R; H223 encoded by CAC is shown (red letters); the guide RNA (5’‐AGGACAGGAACAGGTGCATG‐3’; black letters on yellow background) is shown that targets the TGG protospacer adjacent motif (PAM; yellow letters on gray background) sequence located on the reverse DNA strand. The 75‐bp single‐strand repair template (RT) (gray letters) is shown that replaces the C **A** C codon (His, red letters) with the C **G** A codon (Arg, green letters) at position 223; silent nucleotide changes are indicated (blue letters); the 6‐bp recognition sequence for the endonuclease *Bst*BI (***TT/CG AA***) is underlined.

Generation of mice expressing the PTH1R with an HA‐tag (referred herein as *WT‐PTH1R*) has been described elsewhere^(^ ^27^ ^)^ (Fig. 1, upper panel). To replace histidine (His) at position 223 with arginine (Arg), *improved genome editing* via *Oviductal Nucleic Acids Delivery* (i‐GONAD)^(^ ^28^, ^29^, ^30^ ^)^ was performed approximately 16 h after coitus on wild‐type (WT) females (CD1) that had been mated with a male homozygous for the *WT‐PTH1R* (C57BL6/CD1 mixed strain). Ovaries and oviducts were surgically exposed through dorsolateral incisions. Tip‐tapered glass capillary pipettes were then used to inject into the lumen of each oviduct 2 μl of a premixed genome‐editing CRISPR solution that was prepared by mixing 1.5 μl of the two‐piece guide RNA (crRNA and tracrRNA: final concentration 30 μM; IDT, Coralville, IA, USA), 1.5 μl of a single‐strand DNA repair template comprising 75 nucleotides (3 μg/μl; IDT), which introduced the H223R mutation as well as five silent nucleotide changes (Fig. 1), 1 μl of recombinant Cas9 (4 μg/μl; Sigma‐Aldrich, St. Louis, MO, USA), and 0.4 μl of Fast Green dye (0.1 mg/ml; Sigma‐Aldrich, St. Louis, MO). Immediately after injection, the entire oviduct was covered with a sterile Kimwipe (Kimberly‐Clark, Irving, TX, USA) that had been soaked in PBS, then electroporation was performed using a BTX‐820 square pulse generator with electrode tweezers, CUY652P2.5X4 (Nepa Gene, Chiba, Japan). The pulse generator was set at 50 V, 5 ms, eight pulses, and the electrode gap was 0.5 cm. The ovaries and oviducts were subsequently returned to their original positions and the incisions were closed and sutured. Genome‐edited and nonedited embryos subsequently developed to term and pups were genotyped to determine whether mutations had been introduced into the human *WT‐PTH1R*. As outlined earlier, males used for i‐GONAD were homozygous for the *WT‐PTH1R* while females were homozygous for the mouse WT receptor (*wt‐Pth1r*). Offspring were thus carriers of a maternal *wt‐Pth1r* allele and a paternal *PTH1R* allele that was either WT or mutant, which made it possible to limit the subsequent PCR‐based search for the intended mutation to the “humanized” paternal allele.

### PCR, gel electrophoresis, and nucleotide sequence analysis

Genomic DNA was isolated from mouse tails (age < 20 days) or ears (age > 20 days), as described.^(^ ^23^ ^)^ PCR was performed using 2 μl DNA with forward primer F1 (5′‐ggaagctcTACCCTTACGATGTT‐3′; capital letters indicate nucleotides encoding portions of the HA‐tag) and reverse primer R1 (5′‐aggaagtaaaggaagaaggtcacag‐3′) (Fig. S1 *A*; amplification scheme); cycler protocol: initial denaturing at 95°C for 5 min, followed by 35 cycles of denaturing at 95°C for 15 s, annealing 58°C for 10 s, and 72°C for 20 s; final extension 10 min. After gel electrophoresis (1.6% agarose stained with Gel Green, Biotium, Fremont, CA, Catalog No. 41005), DNA bands of the expected size (622 bp) were excised, purified, and submitted for nucleotide sequence analysis at the MGH sequencing core using amplification primers. To determine whether the A > G nucleotide change, which introduces the H223R mutation, was present, PCR amplicons were incubated with *Bst*B1 (30 min, 65°C) before performing another gel electrophoresis.

### Micro–CT (μCT) analyses

Assessment of bone morphology was performed by the Center for Skeletal Research Imaging and Biomechanical Testing Core using μCT (μCT40, Scanco Medical, Brüttisellen, Switzerland). Dissected femura isolated from F1 *H223R‐PTH1R/wt‐Pth1r* offspring or *wt‐Pth1r/wt‐Pth1r* littermates at postnatal day 42 were scanned with a 10‐μm isotropic voxel size, 70 kV peak potential (kVp), 114 μA X‐ray tube intensity, and 200 ms integration time. The samples were segmented with a threshold of 350 mg HA/cm^3^ to create three‐dimensional reconstructions.

### Blood marker analyses

Blood was collected from mice by cardiac puncture for analysis of several biochemical parameters. Serum total calcium was determined using Stanbio Laboratory Calcium Liquidcolor (CPC; Boerne, TX, USA; Catalog No. 0150‐250). Serum inorganic phosphate was determined using a Phosphate Colorimetric Assay Kit (BioVision, Inc., Milpitas, CA, USA; Catalog No. K410‐500). Serum C‐terminal telopeptides of types I and II collagen (CTX‐1 and CTX‐2; Immunodiagnostic Systems Inc., Fountain Hills, AZ, USA; Catalog No. AC‐06F1 and AC‐08F1, respectively), as well as N‐terminal propeptide of type I procollagen (P1NP) were determined using the RatLaps EIA kit (Immunodiagnostic Systems Inc., Fountain Hills, AZ, USA; Catalog No. AC‐33F1). Serum intact mouse parathyroid hormone (PTH(1‐84)) was measured using the mouse PTH(1‐84) EIA (Quidel Corp, San Diego, CA, USA; Catalog No. 60‐2305).

### Statistical analyses

To determine whether differences in the different blood markers were significantly different, the nonparametric Mann–Whitney test was used. Differences at *p* < 0.05 were considered significant.

### Histology, TUNEL staining, and RNAscope

Femura and tibiae were harvested from F1 *H223R‐PTH1R/wt‐Pth1r* males and females on postnatal days 6, 12, and 27, along with samples from their *wt‐Pth1r/wt‐Pth1r* littermates. After fixation with 10% formalin for 2 days, bones were rinsed with PBS before being transferred to 70% EtOH. Sections (5 μm) were stained with hematoxylin and eosin (H&E) and analyzed by light microscopy (Keyence BZ‐X710). Terminal deoxynucleotidyl transferase dUTP nick end labeling (TUNEL) staining was performed on paraffin sections using an in situ Cell Death Detection kit (Roche, Mannheim, Germany; Catalog No. 11684795910), as previously reported.^(^ ^31^ ^)^ RNA in situ hybridization analysis was carried out on adjacent sections using the RNAscope 2.5 HD Detection Reagent ‐ RED kit (Advanced Cell Diagnostics, Hayward, CA; Catalog No. 322360); the same company provided the probes specific for mRNAs derived from *Col2a1* (Catalog No. 407221), *Col10a1* (Catalog No. 426181) or *PTH1R* (Catalog No. 537131).^(^ ^32^ ^)^

## Results

### i‐GONAD to generate mice expressing PTH1R mutants

To generate a JMC model that recapitulates the human disease more faithfully than previously generated transgenic mice,^(^ ^24^, ^25^, ^26^ ^)^ we used i‐GONAD^(^ ^28^, ^29^, ^30^ ^)^ to introduce the H223R mutation into zygotes from WT CD1 females that had been mated approximately 16 h earlier with a male homozygous for the human *PTH1R*.^(^ ^27^ ^)^ The guide RNA targeted a protospacer adjacent motif (PAM) sequence close to the codon for residue 223 of the *PTH1R* to cut the double‐stranded target DNA with recombinant Cas9 protein before introducing through a repair template the H223R mutation as well as several silent nucleotide changes (Fig. 1). Three independent i‐GONAD procedures resulted in three litters with 25 pups in total (Table S1) that were expected to carry the WT or a mutated human *PTH1R* on the paternal allele. Consequently, PCR amplification of genomic DNA using primers specific for the human allele yielded the predicted 622‐bp band for all F0 pups (examples shown in Fig. S1 *A*; uncut, left gel).

### Most pups generated through i‐GONAD lack the H223R mutation and thus revealed no phenotype

Fifteen of the 25 offspring showed no obvious physical abnormality, and their PCR amplicons remained intact after incubation with *Bst*BI, indicating an absence of the H223R mutation (example shown in Fig. S1 *A*, *Bst*BI, lane −). For eight of these pups, nucleotide sequence analysis of the amplicons revealed only the unmodified *WT‐PTH1R* (example shown in Fig. S1 *B*, upper panel)^(^ ^27^ ^)^ or mosaicism for the unmodified *WT‐PTH1R* allele in combination with a *PTH1R* allele carrying an unintended deletion or insertion (see following discussion).

### Early lethality of F0 founders that were heterozygous for the H223R‐PTH1R mutant

PCR amplicons from several pups yielded, after incubation with *Bst*BI, DNA bands of 413 and 209 bp, that is, sizes expected for the human PTH1R with the H223R mutation, but no evidence for an undigested 622‐bp band derived from the WT‐PTH1R (example shown in Fig. S1 *A*, *Bst*BI, lane +). Nucleotide sequence analysis of these PCR products revealed only the DNA sequence that had been introduced through the repair template, including the nucleotide change that replaces the codon for histidine 223 with that for arginine (H223R mutation), as well as the five silent nucleotide changes (example shown in Fig. S1 *B*, middle panel; see also Table S1). This indicated that these pups were nonmosaic, that is, derived from zygotes that had undergone genome editing at the single‐cell stage. These pups exhibited obvious phenotypic abnormalities by the end of the first week of life that included outwardly rotated and flattened paws and much reduced mobility. These offspring failed to gain weight, showed paradoxical breathing suggestive of ribcage instability and, thus, reduced functionality of the diaphragm, and they died between postnatal days 10 and 60, possibly because of respiratory compromise. Craniofacial features appeared grossly normal, although the pups that survived longer showed a delay in the eruption of lower and upper incisors. One female with the H223R mutation survived until day 60, but mating attempts were unsuccessful.

### Prolonged survival of F0 founders that are mosaic for the H223R mutation and a second PTH1R allele

DNA from four additional pups generated PCR products that were cut incompletely by *Bst*BI and thus revealed a 622‐bp DNA band as well as 413‐ and 209‐bp fragments (example shown in Fig. S1 *A*; *Bst*BI, lane m). Starting at the position of the first silent nucleotide change, nucleotide sequence analyses revealed two overlapping nucleotide sequences as evidence for the presence of two distinct alleles, namely, one allele in which the repair template had introduced the intended six nucleotide changes (H223R plus the five silent nucleotide changes) and a second allele with either an insertion or a deletion, for example, a deletion of 10 nucleotides that resulted in a shift of the open‐reading frame followed by a premature termination codon (example shown in Fig. S1 *B*, lower panel).

Two mosaic males carried the *H223R‐PTH1R* allele in some cells and, in other cells, either an insertion of 36 nucleotides after the H223 codon (male no. 8) or a deletion of the six nucleotides that encode amino acids 225 and 226 (*delH225/L226‐PTH1R*, *PTH1R‐del*; male no. 9). The in‐frame insertion or deletion alleles lacked the H223R mutation and are expected to encode PTH1R variants that are hypomorphic or nonfunctional. Both of these mosaic founders developed obvious phenotypic changes later in life but lived for several months and were fertile (Table S1).

### Mosaic male founders sired F1 pups heterozygous for the H223R mutation or PTH1R with 6‐bp deletion

The mosaic male founders nos. 8 and 9 (mixed CD1/C57BL6 background) allowed the generation of F1 offspring that were expected to be either homozygous for two WT mouse alleles (genotype: *wt‐Pth1r/wt‐Pth1r*) or heterozygous for the WT mouse *Pth1r* allele combined with one of three possible human *PTH1R* alleles, namely, the allele with the H223R mutation alone (genotype: *H223R‐PTH1R/wt‐Pth1r*), the allele with the 36‐bp insertion (genotype: *PTH1R‐insert/wt‐Pth1r* from founder no. 8), or the allele with the 6‐bp deletion (genotype: *PTH1R‐del/wt‐Pth1r* from founder no. 9).

The F1 pups carrying the H223R allele derived from either founder no. 8 or 9 males died within ~40 days of birth and did not mate. Matings were performed with WT females in genetically different strains, including CD1, C57BL6, Friend Virus B (FVB), and the “humanized” *PTH1R* (CD1/C57BL6); the FVB and the “humanized” *PTH1R* background provided no obvious survival advantage of F1 pups heterozygous for H223R; matings for histological studies and laboratory measurements were therefore pursued only with CD1 and C57BL6 females. In total, 101 F1 pups were obtained from mating mosaic males no. 8 or 9 with either CD1 (42 pups) or C57BL6 (59 pups) females. Of these, 51 mice (30 males, 21 females) had no obvious phenotype, their genomic DNA yielded no PCR amplification when using *PTH1R*‐specific primers, and so they were considered to be homozygous for the WT mouse *Pth1r* allele (*wt‐Pth1r/wt‐Pth1r*; Table S2). Two pups that were sired by mosaic male no. 9 appeared normal, yet these were found to carry the “humanized” *PTH1R* allele comprising the 6‐bp deletion. The remaining 48 pups (30 males, 18 females) showed an obvious phenotype and were found to be carriers of the H223R mutation. The *PTH1R* allele with the 36‐bp insertion was not observed in any of the F1 pups sired by mosaic male no. 8 that had shown low mosaicism for this mutation, as determined by nucleotide sequence analysis as well as *Bst*BI digestion of the PCR product derived from the genomic DNA of this male. It was therefore not surprising that pups from the four litters sired by male no. 8 were either homozygous *wt‐Pth1r/wt‐Pth1r* or *H223R‐PTH1R/wt‐Pth1r* but showed no *PTH1R‐insert/wt‐Pth1r* allele.

**Fig. 2:**
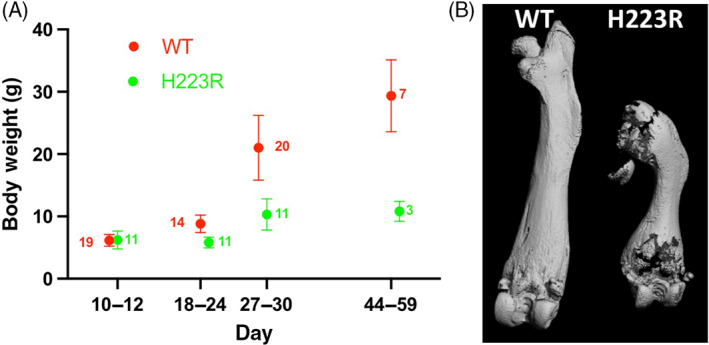
Weight gain and micro‐CT images of F1 *H223R‐PTH1R/wt‐Pth1r* (H223R) mice in comparison to their WT littermates (*wt‐Pth1r/wt‐Pth1r*, WT). All pups were derived from matings between mosaic male founders (mixed CD1/C57BL6) and CD1 females. (*A*) Weight curves of F1 *H223R‐PTH1R/wt‐Pth1r* (H223R) pups and their littermates that are homozygous *wt‐Pth1r/wt‐Pth1r* (WT). The number of pups in each age group is indicated. (*B*) Micro‐CT analyses of femora from F1 *H223R‐PTH1R/wt‐Pth1r* (H223R) mouse and its homozygous *wt‐Pth1r/wt‐Pth1r* (WT) littermate control (both are male littermates; postnatal day 42).

F1 *H223R‐PTH1R* pups showed reduced weight gain by the third week of life and little or no increase in weight thereafter (Fig. 2*A*), and, like the nonmosaic *H223R‐PTH1R* founders, they showed paradoxical breathing suggestive of impaired ribcage stability. A μCT image that was obtained of a femur from a 42‐day‐old male mouse with the H223R mutation showed a markedly reduced length and an abnormal shape, as compared to the same bone from a homozygous *wt‐Pth1r/wt‐Pth1r* male littermate of the same age (Fig. 2*B*).

### Laboratory findings in F1 mice heterozygous for H223R mutation and homozygous wt‐Pth1r littermate controls

Some of the *H223R‐PTH1R/wt‐Pth1r* pups died by 3 weeks of age prior to collection of blood samples, while other animals with the same genotype showed no obvious distress for several weeks other than abnormal breathing. The majority of blood samples was obtained at sacrifice between days 28 and 60 from *H223R‐PTH1R/wt‐Pth1r* pups and their homozygous *wt‐Pth1r/wt‐Pth1r* control littermates (males and females); these were mostly offspring from WT CD1 females as this strain was used predominantly after other backgrounds were shown to provide no survival advantage. Blood samples were also obtained from three H223R mice on day 72, one on day 97, and two on day 105, along with samples from their control littermates.

**Fig. 3:**
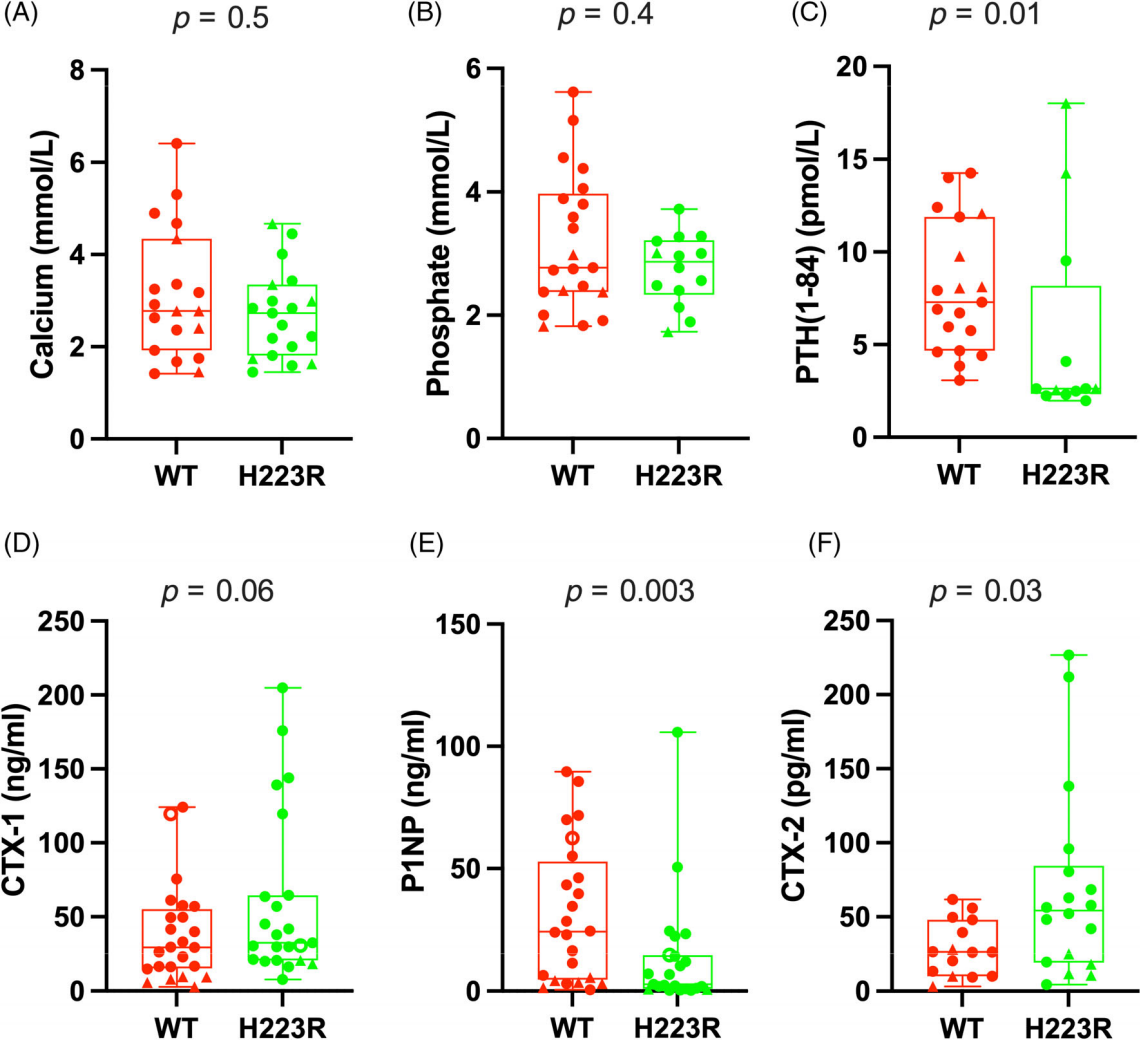
Serum measurements in F1 *H223R‐PTH1R/wt‐Pth1r* (H223R) mice in comparison to their homozygous *wt‐Pth1r/wt‐Pth1r* littermate controls (WT). All matings were between mosaic male no. 9 with either CD1 and C57BL6 females. Filled circles, CD1 males; filled triangles, CD1 females; open circles, C57BL6 males. Conversion factors (mmol/L to mg/dl): calcium multiply by 4 and phosphate multiply by 3.1; PTH (pmol/L to pg/ml) multiply by 10. The *p* values for statistical differences between both groups were calculated by nonparametric Mann–Whitney test.

Total serum calcium and phosphate levels, males and females combined, were not statistically different when comparing the results for mice homozygous for the *wt‐Pth1r/wt‐Pth1r* allele to those with the *H223R‐PTH1R/wt‐Pth1r* allele (Fig. 3*A*,*B* and Table S3). However, the average blood concentrations of PTH, P1NP, and CTX‐2 were significantly different for WT and mutant mice, while the differences for CTX‐1 levels did not reach significance. PTH levels were at or below the lowest PTH(1‐84) standard (28 pg/ml = 2.8 pmol/L) for most animals with the H223R allele, so they were significantly below the mean PTH level in control littermates homozygous for the *wt‐Pth1r* allele (Fig. 3*C* and Table S3). The average level of serum CTX‐1, which assesses the rate of bone resorption, tended to be higher in mice heterozygous for the H223R mutation than for *wt‐Pth1r/wt‐Pth1r* controls (Fig. 3*D* and Table S3). The average blood level of P1NP, a marker of bone formation, was significantly lower in the H223R mutants than in the homozygous *wt‐Pth1r* controls (Fig. 3*E* and Table S3). The average level of blood CTX‐2, a marker of type II collagen breakdown,^(^ ^33^ ^)^ was higher in mice heterozygous for the H223R mutation than in mice homozygous for *wt‐Pth1r*. Compared to the results that combined data for males and females, differences were more pronounced when comparing only the measurements of males that had the genotype *wt‐Pth1r/wt‐Pth1r* or *H223R‐PTH1R/wt‐Pth1r* (Table S3). In fact, analysis of samples from females alone revealed no statistically significant differences for all six biochemical parameters, which is most likely related to the limited number of available samples from females, rather than gender difference.

### Growth plate abnormalities in F1 mice with heterozygous H223R‐PTH1R mutation and assessment of chondrocyte mRNA marker expression

To assess the expected progression of growth plate abnormalities caused by the constitutively active H223R‐PTH1R mutant, a WT CD1 female was mated with mosaic male no. 9 (heterozygous for *wt‐Pth1r/*mosaic for *H223R‐PTH1R* and *PTH1R‐del*). This litter provided 14 pups that were either homozygous *wt‐Pth1r/wt‐Pth1r* (*n* = 6), *H223R‐PTH1R/wt‐Pth1r* (*n* = 7), or *PTH1R‐del/wt‐Pth1r* (*n* = 1). Pups were sacrificed on postnatal day 6, 12, or 27, and their right tibiae were collected for analyses.

**Fig. 4:**
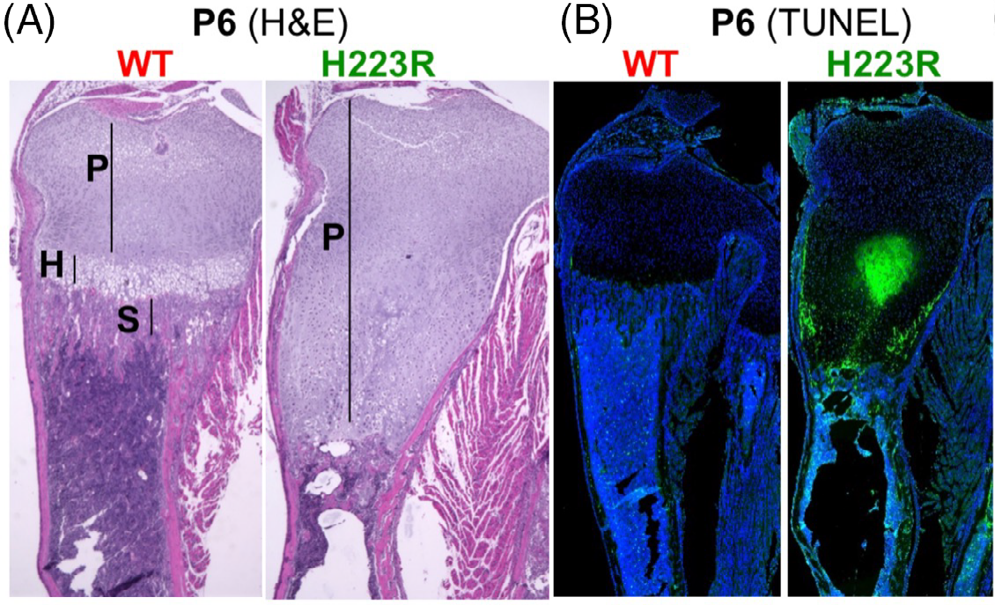
H&E and TUNEL staining of WT and mutant H223R mice on postnatal day 6. Mating between mosaic male no. 9 with a CD1 WT female. Tibiae from F1 littermates that are *H223R‐PTH1R/wt‐Pth1r* (H223R, male) or homozygous *wt‐Pth1r/wt‐Pth1r* (WT, female). P, proliferating chondrocytes; H, hypertrophic chondrocytes; S, primary spongiosa.

On postnatal day 6, H&E staining of tibiae from pups that were homozygous for the *wt‐Pth1r* revealed normal growth plates with orderly aligned columns of proliferating and hypertrophic chondrocytes and adjacent well‐demarcated surfaces of primary spongiosa (Fig. 4, left panels). Tibial growth plates from the *H223R‐PTH1R* pups were strikingly different and showed a layer of proliferating chondrocytes that was at least twice as wide as that in WT growth plates, there was no defined layer of hypertrophic chondrocytes, and there was no defined surface of nascent bone at the sites where the primary spongiosa should form. TUNEL analysis of tibiae from homozygous *wt‐Pth1r* mice showed a few positive cells in the proximity of the primary spongiosa, while tibiae from *H223R‐PTH1R/wt‐Pth1r* mice showed a much larger number of TUNEL‐positive (dying) cells that were localized in the inner regions of the growth plates.

**Fig. 5:**
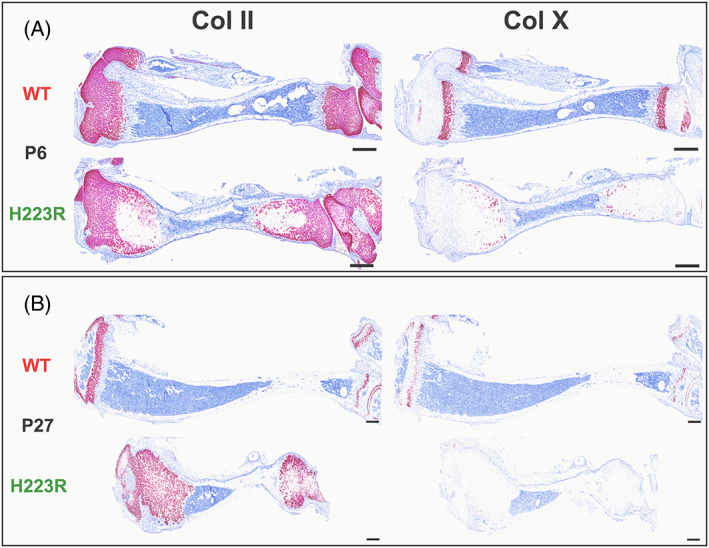
RNAscope for type II (*A*) and type X collagen (*B*) on postnatal days 6 and 27: mating between mosaic male no. 9 with a CD1 WT female. Tibiae from F1 *H223R‐PTH1R/wt‐Pth1r* pups (H223R, male) and homozygous *wt‐Pth1r/wt‐Pth1r* littermate controls (WT, male). Equivalent results were obtained for tibiae from *wt‐Pth1r/wt‐Pth1r* and *H223R‐PTH1R* pups on day 12 (data not shown). Scale bar, 3 mm.

Tibiae of 6‐day‐old pups with the H223R mutation contained a zone of type II collagen‐positive chondrocytes that was markedly expanded relative to that seen in tibiae from the *wt‐Pth1r/wt‐Pth1r* littermate controls (Fig. 5*A*). On postnatal day 27, this zone was increased in size in the H223R tibiae, relative to day 6, whereas it was decreased considerably in size in the WT bones. WT tibiae furthermore showed a well‐defined layer of collagen type X‐positive hypertrophic chondrocytes on postnatal day 6, which decreased in width by day 27, while tibiae from the *H223R‐PTH1R* mice showed only a few scattered chondrocytes that were positive for collagen type X expression (Fig. 5*B*). The bone marrow space was also much reduced in the bones of the H223R mutant mice as compared to those of WT mice at postnatal days 6 and 27.

**Fig. 6:**
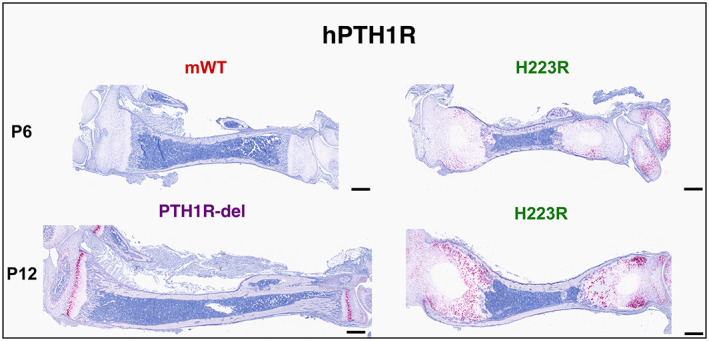
RNAscope for human *PTH1R*: mating between mosaic male no. 9 with a CD1 WT female. Tibiae from F1 pups that were *H223R‐PTH1R/wt‐Pth1r* (H223R, males), homozygous *wt‐Pth1r/wt‐Pth1r* (mouse WT, male), or *PTH1R‐del/wt‐Pth1r* (PTH1R‐del, male). RNAscope on postnatal days 6 and 12 for human *PTH1R*. The human *PTH1R* mRNA was not detected in tibiae from *wt‐Pth1r/wt‐Pth1r* pups, but staining was obtained for the tibiae from *H223R‐PTH1R/wt‐Pth1r* pups on days 6 and 12 and for the tibia from a *PTH1R‐del/wt‐Pth1r* pup on day 12. Detection of mutant PTH1R was similar in *H223R‐PTH1R/wt‐Pth1r* pups on day 27 (data not shown). Scale bar, 3 mm.

As expected, in situ hybridization analysis using probes specific for the human *PTH1R* sequence did not detect a signal in the tibiae of mice homozygous for the *wt‐Pth1r* allele on postnatal day 6 (as well as on day 27, not shown), whereas positive staining was observed in the tibia from a mouse that was heterozygous for a human PTH1R allele with a 6‐bp deletion (*PTH1R‐del/wt‐Pth1r*; postnatal day 12) (Fig. 6). These PTH1R‐positive cells formed a narrow band at the layer where chondrocytes transition from proliferative to hypertrophic cells right above the layer of collagen type X‐positive cells, a location consistent with prior PTH1R mRNA expression data obtained in rodent tibiae.^(^ ^11^ ^)^ In the H223R mutant tibiae, *PTH1R* mRNA expression was not localized to a defined band of cells but rather was scattered throughout the growth plate, in a pattern similar to that seen for collagen type X expression, and overlapping at least partly with the broad layer of prehypertrophic chondrocytes expressing collagen type II mRNA (Fig 6).

Taken together, these findings are consistent with the notion that expression of a constitutively active H223R‐PTH1R mutant in the growth plates delays or prevents chondrocyte hypertrophy and thus alters the overall process by which the cartilage template is formed and ultimately replaced by mineralized bone.

## Discussion

Expression of the murine *Pth1r* with the H223R mutation in COS‐7 cells had previously revealed much lower constitutive activity than is observed with cells expressing the human PTH1R with this mutation.^(^ ^6^ ^)^ Consistent with this limited in vitro phenotype, preliminary studies in mice heterozygous for the H223R mutation, introduced into the mouse *Pth1r* gene through a traditional gene‐targeting approach, had revealed only a mild and transient delay in chondrocyte maturation.^(^ ^34^ ^)^ Because of enhanced agonist‐independent cAMP formation by the H223R‐PTH1R when tested in vitro, we hypothesized that the H223R mutation, the most frequent genetic cause of JMC,^(^ ^5^ ^)^ would lead to a more pronounced growth plate phenotype when introduced into the “humanized” mouse that was recently generated to express the human PTH1R under the control of the endogenous murine promoter.^(^ ^27^ ^)^

Previous transgenic mice expressing the *H223R‐PTH1R* mutant under the control of a collagen type Ia1 promoter (C1‐HR), which targets the mutant GPCR to osteoblasts, exhibited a phenotype that was, not surprisingly, quite different from that seen in JMC patients. In fact, these C1‐HR mice show robust excess bone formation that fills nearly the entire marrow compartment, coupled with cortical bone thinning and no change in mineral ion homeostasis.^(^ ^23^, ^24^ ^)^ Twice daily treatment of C1‐HR mice with a PTHrP(7‐36) peptide inverse agonist analog (PTH‐IA) led to a significant reduction in bone mass, but not to full normalization of the skeletal abnormalities. Very similar findings were also obtained when the PTH‐IA was infused for 2 weeks by minipump (unpublished data). That only partial efficacy was achieved in these studies likely reflects a combination of different factors, including a limited capacity of the peptide to suppress the receptor's constitutive activity,^(^ ^5^, ^20^ ^)^ a short half‐life of the peptide in the circulation, and the likely high expression levels of the constitutively active mutant PTH1R due to the use of the heterologous collagen type Ia1 promoter.^(^ ^23^ ^)^ We thus sought to generate a mouse that recapitulates more accurately the human disease by expressing the mutant PTH1R protein in all tissues that normally respond to PTH or PTHrP, and at normal endogenous levels, and consequently introduced the H223R mutation into the “humanized” *PTH1R* mouse.^(^ ^27^ ^)^ This was accomplished through the i‐GONAD approach, which enables the development of genetically manipulated knock‐in mice within weeks, as opposed to months or years, as typically needed when using traditional gene‐targeting approaches.^(^ ^29^ ^)^ Furthermore, for mutations that potentially result in a lethal phenotype, the i‐GONAD approach has the advantage of generating F0 founder mice that show different levels of mosaicism and, thus, improved survival.^(^ ^28^ ^)^ It may then be possible to breed such mosaic founders and, if F1 pups with the intended mutation are identified, to characterize the phenotype and explore the cause of lethality.

We found that most of the F0 mice obtained from the i‐GONAD procedure had incorporated the *H223R‐PTH1R* allele and revealed a severe postnatal phenotype. These animals failed to gain weight after 2 weeks of life and typically died before 2 months of age without producing offspring. However, we identified several mosaic F0 *H223R‐PTH1R* mice that showed improved survival and could be mated to produce several litters of nonmosaic F1 H223R heterozygous pups, which allowed us to assess the impact of the mutation on growth plate cartilage and mineral ion physiology. Although we did not detect a statistically significant difference in serum calcium and phosphate levels between the H223R‐PTH1R mice and their *wt‐Pth1r/wt‐Pth1r* littermates, serum PTH(1‐84) levels were notably suppressed and below or close to the lowest assay standard (28 pg/ml = 2.8 pmol/L) for the H223R mice, as compared to the WT mice, for which serum PTH levels were within the normal range. This apparent reduction in PTH levels in mice with the H223R mutation is fully consistent with the findings in JMC patients^(^ ^5^ ^)^ and likely reflects a reduced need for circulating PTH to maintain normal blood calcium homeostasis when the constitutively active H223R‐PTH1R mutant is expressed in bone and kidney. It therefore seems plausible that blood calcium levels are only intermittently and/or minimally elevated in the H223R‐PTH1R mice, as is also seen in older and most newborn JMC patients.^(^ ^5^ ^)^

The slight, nonsignificant elevation in serum CTX‐1 observed in the heterozygous *H223R‐PTH1R* mice is consistent with an increase in bone resorption, while the statistically significant reduction in serum P1NP is consistent with a delay in bone mass accrual. Given the markedly increased zone of collagen type II‐positive cells observed in the growth plates of the H223R mice, we also assessed serum CTX‐2 levels as a potential indirect indicator of the activity and/or proliferation of growth plate chondrocytes, as collagen type II presumably undergoes cleavage within the growth plates. We found the circulating serum CTX‐2 levels to be moderately elevated in the H223R mice, as compared to WT controls, which supports the notion that this breakdown product may be useful as a biomarker for tracking changes in the growth plate chondrocytes during therapeutic interventions.

In comparison to the WT animals, the tibiae of the mutant H223R mice were considerably shorter and had reduced bone marrow spaces. Analysis of the growth plates revealed no defined columns of hypertrophic chondrocytes and virtually no primary spongiosa, which is remarkably similar to the growth plate findings in a recently reported JMC patient, although the age of that patient and the identified *PTH1R* mutation had not been disclosed.^(^ ^35^ ^)^ *H223R‐PTH1R* mice showed already by postnatal day 6, a major enlargement of the zone of proliferating chondrocytes that abundantly express type II collagen. Strikingly, the inner region of this expanded zone was devoid of cells expressing the mRNA encoding this collagen. Instead, these interior cells exhibited strong positive staining for TUNEL, indicating increased cell death, which was not observed in the interior regions of the growth plates in *wt‐Pth1r*/*wt‐Pth1r* littermate controls. The reason for the enhanced cell death seen in the H223R growth plates is unknown but may involve, at least in part, a reduction in the diffusion of oxygen into the central area, given the substantial expansion in size of this intrinsically avascular zone. Indeed, mice having conditional knockout mutations of HIF1a that result in impaired adaptation to hypoxia in growth plate chondrocytes exhibit a similar phenotype of cell death in the central chondrocyte zone.^(^ ^36^, ^37^ ^)^ It will be interesting to investigate whether and how hypoxia‐driven metabolic pathways contribute to the abnormalities seen in the developing growth plates of the *H223R‐PTH1R* mutant mice, as this aspect of the cell and molecular changes that occur in growth plates has not yet been explored for JMC patients.

The lack of well‐organized columns of type X collagen‐positive hypertrophic chondrocytes, in combination with dead or dying chondrocytes in the central portion of the growth plates, likely contributes to the impaired growth of long bones in *H223R‐PTH1R* mice. We note that abundant collagen type II and diminished collagen type X expression in these mice is opposite to the findings in mice that are null for *Pthlp* (encoding PTHrP) or *wt‐Pth1r*,^(^ ^38^, ^39^ ^)^ both of which show a major acceleration of the transition from proliferative to hypertrophic chondrocytes. The results with *H223R‐PTH1R* mice are, however, very similar to those in mice lacking SIK3 or SIK2/3 in the growth plates,^(^ ^40^ ^)^ which is consistent with the critical roles that these kinases play downstream of PTH1R and PKA signaling during bone development. Similar observations were not made in C2‐HR transgenic animals expressing the H223R‐PTH1R protein in growth plates via the collagen type 2 promoter.^(^ ^26^ ^)^ This indicates that the C2‐HR transgene does not recapitulate the physiological spatiotemporal expression pattern of the mutant H223R‐PTH1R protein and, consequently, does not generate cAMP at levels that are sufficient to delay the differentiation process by which chondrocytes transit from proliferation to hypertrophy.

Given the profound abnormalities observed on day 6 of life, it is likely that growth plate abnormalities can be observed at postnatal day 1 or even earlier in the *H223R‐PTH1R* mice. This raises the question of whether early and prolonged treatment with a PTH inverse agonist peptide, such as the [D‐Trp12]PTHrP(7‐36) analog used previously in C1‐HR mice,^(^ ^23^ ^)^ can lead to improved skeletal growth and development in these mice. Ideally, to be effective in JMC patients, such a treatment would, in addition to improving skeletal growth,^(^ ^5^ ^)^ improve also overall mineral ion homeostasis and prevent, or at least limit, the risk of nephrocalcinosis and a progressive decline in renal function. Studies to help address these challenges could be pursued using *PTH1R‐H223R* mice, which were designed to express the mutant allele in all relevant PTH and PTHrP target tissues and at the proper time and cell surface levels, as occurs in patients.

In summary, i‐GONAD was used to introduce the H223R‐PTH1R mutation, the most frequent cause of JMC, into “humanized” *PTH1R* mice, thereby allowing proper spatial and temporal expression through the endogenous mouse *Pth1r* gene promoter. The mutation resulted in a severe phenotype, not seen in previously generated transgenic JMC mouse models, that is characterized by a lack of weight gain, early lethality, impaired respiratory function, and severe skeletal defects. The growth plates of heterozygous F1 *H223R‐PTH1R* pups showed markedly expanded zones of proliferating chondrocytes, central cores with increased chondrocyte death, a lack of organized columns, few and dispersed hypertrophic chondrocytes, and no primary spongiosa. These mice could provide a suitable model for exploring the efficacy of candidate treatment modalities for JMC intended to help mitigate the skeletal and renal complications of this disease and for developing biomarkers to monitor such interventions.

## Author Contributions

Monica Reyes: Data curation; Visualization; Methodology; Investigation; Writing ‐ review & editing; Conceptualization. Damla Firat: Data curation; Formal analysis; Writing ‐ review & editing; Methodology. Patrick Hanna: Writing ‐ review & editing; Investigation; Methodology; Conceptualization. Mohd Khan: Data curation; Formal analysis; Writing ‐ review & editing; Methodology; Investigation; Visualization. Michael Bruce: Data curation; Formal analysis; Visualization; Methodology; Investigation; Writing ‐ review & editing. Maria Shvedova: Data curation; Methodology; Investigation; Writing ‐ review & editing. Tatsuya Kobayashi: Conceptualization; Data curation; Formal analysis; Methodology; Investigation; Supervision; Writing ‐ review & editing; Resources. Ernestina Schipani: Conceptualization; Data curation; Formal analysis; Methodology; Investigation; Supervision; Writing ‐ review & editing; Resources. Thomas J. Gardella: Conceptualization; Data curation; Formal analysis; Methodology; Investigation; Supervision; Writing ‐ review & editing; Project administration; Funding acquisition; Writing ‐ original draft; Validation; Resources. Harald Jüppner: Conceptualization; Data curation; Formal analysis; Writing ‐ original draft; Methodology; Investigation; Supervision; Project administration; Writing ‐ review & editing; Funding acquisition; Validation; Resources.

## Disclosures

None of the authors has potential conflicts of interest.

### Peer Review

The peer review history for this article is available at https://www.webofscience.com/api/gateway/wos/peer‐review/10.1002/jbm4.10802.

## Supporting information


**Fig. S1.** Genotyping of i‐GONAD‐generated pups by PCR.
**Table S1.** Genetic and phenotypic characteristics of pups from three litters obtained after mating homozygous “humanized” *PTH1R* (WT‐PTH1R) males with WT CD1 females (wt‐Pth1r) that underwent i‐GONAD approximately 16 h after coitus. All offspring thus carry one *wt‐Pth1r* allele and one WT or genetically altered *PTH1R* allele. Shown are the number of pups without a phenotype that were heterozygous for *WT‐PTH1R*, *WT‐PTH1R* plus an insertion or a deletion (indel), or a *PTH1R* with an indel alone, and the number of pups with an obvious phenotype that were either heterozygous for *H223R‐PTH1R* alone or mosaic for *H223R‐PTH1R* and an indel; two pups had died by 10 days of age and their carcasses were inadvertently discarded by the animal facility, so no tissue was available for DNA extraction.
**Table S2.** Mosaic male founders nos. 8 and 9 were mated with WT CD1 or C57/BL6 females. This resulted in male or female offspring that were *wt‐Pth1r/wt‐Pth1r, PTH1R‐del/wt‐Pth1r*, or *H223R‐PTH1R/wt‐Pth1r*. Matings with CD1 females (*n* = 7) resulted in 45 pups with the H223R mutation and 39 pups homozygous for *wt‐Pth1r*. Matings with C57/BL6 females (*n* = 2) resulted in six pups with the H223R mutation and nine pups homozygous for the *wt‐Pth1r*; the 2 *PTH1R‐del/wt‐Pth1r* pups were from mating no. 9 with a CD1 female.
**Table S3.** Laboratory findings in male and female mice that are either *wt‐Pth1r/wt‐Pth1r* or *H223R‐PTH1R/wt‐Pth1r*. Mean, SD, and SEM, as well as median, number of animals/group, and the *p* values for differences between WT and mutant mice were calculated by nonparametric Mann–Whitney test. Conversion factors (mmol/L to mg/dl): calcium multiply by 4 and phosphate multiply by 3.1; PTH (pmol/L to pg/ml) multiply by 10.
