# Supplementary material for: Substantially Delayed Maturation of Growth Plate Chondrocytes in “Humanized” PTH1R Mice with the H223R Mutation of Jansen's Disease
Source: JBMR Plus. 2023 Aug 15;7(10):e10802. doi: 10.1002/jbm4.10802 (PMC10556264; doi:10.1002/jbm4.10802)
Supplement: Supplementary file 1 — Fig. S1. Genotyping of i‐GONAD‐generated pups by PCR. Table S1. Genetic and phenotypic characteristics of pups from three litters obtained after mating homozygous “humanized” PTH1R (WT‐PTH1R) males with WT CD1 females (wt‐Pth1r) that underwent i‐GONAD approximately 16 h after coitus. All offspring thus carry one wt‐Pth1r allele and one WT or genetically altered PTH1R allele. Shown are the number of pups without a phenotype that were heterozygous for WT‐PTH1R, WT‐PTH1R plus an insertion or a deletion (indel), or a PTH1R with an indel alone, and the number of pups with an obvious phenotype that were either heterozygous for H223R‐PTH1R alone or mosaic for H223R‐PTH1R and an indel; two pups had died by 10 days of age and their carcasses were inadvertently discarded by the animal facility, so no tissue was available for DNA extraction. Table S2. Mosaic male founders nos. 8 and 9 were mated with WT CD1 or C57/BL6 females. This resulted in male or female offspring that were wt‐Pth1r/wt‐Pth1r, PTH1R‐del/wt‐Pth1r, or H223R‐PTH1R/wt‐Pth1r. Matings with CD1 females (n = 7) resulted in 45 pups with the H223R mutation and 39 pups homozygous for wt‐Pth1r. Matings with C57/BL6 females (n = 2) resulted in six pups with the H223R mutation and nine pups homozygous for the wt‐Pth1r; the 2 PTH1R‐del/wt‐Pth1r pups were from mating no. 9 with a CD1 female. Table S3. Laboratory findings in male and female mice that are either wt‐Pth1r/wt‐Pth1r or H223R‐PTH1R/wt‐Pth1r. Mean, SD, and SEM, as well as median, number of animals/group, and the p values for differences between WT and mutant mice were calculated by nonparametric Mann–Whitney test. Conversion factors (mmol/L to mg/dl): calcium multiply by 4 and phosphate multiply by 3.1; PTH (pmol/L to pg/ml) multiply by 10. [file JBM4-7-e10802-s001.pdf]

Suppl. Fig. 1

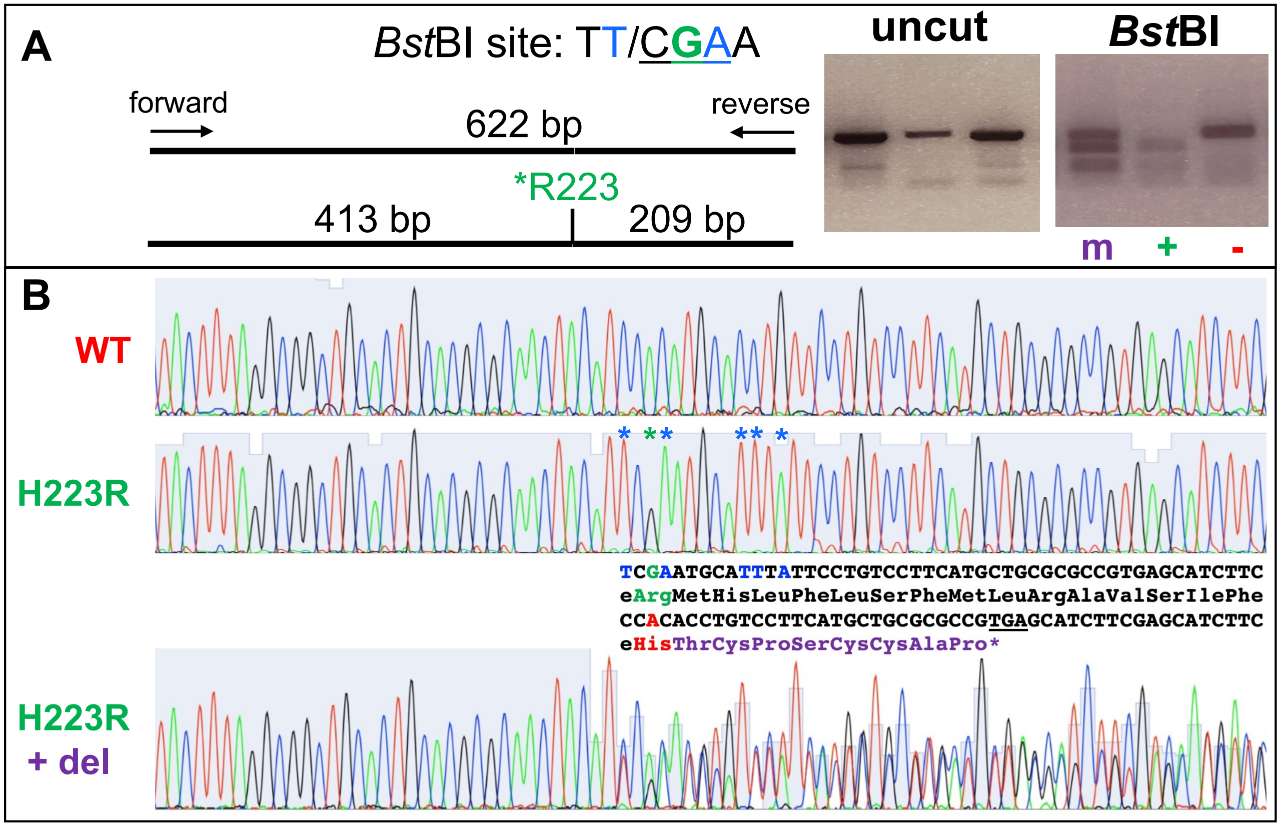

A
BstBI site: TT/CGAA
forward
622 bp
reverse
*R223
413 bp
209 bp
uncut
BstBI
m
+
-
B
WT
* ** ** *
H223R
TCGAATGCATTTATTCCTGTCCTTCATGCTGCGCGCCGTGAGCATCTTC
eArgMetHisLeuPheLeuSerPheMetLeuArgAlaValSerIlePhe
CCACACCTGTCCTTCATGCTGCGCGCCGTGAGCATCTTCGAGCATCTTC
eHisThrCysProSerCysCysAlaPro*
H223R
+ del

Suppl. Table 1: Homozygous "humanized" *PTH1R* males to generate *H223R-PTH1R* founders through i-GONAD

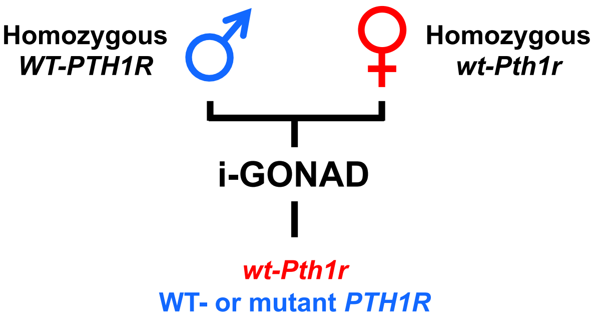

Human *PTH1R* genotypes	Litter 1 n=10	Litter 2 n=11	Litter 3 n=4	n	Phenotype	Age at death
WT alone	1	7	0	8	no	culled
Mosaic WT plus an insertion or deletion	0	1	1	2	no	culled
Only insertion or deletion	4	0	1	5	no	culled
**H223R only**	**2**	**2**	**0**	**4**	**yes[+]**	**10-60 days[+++]**
**Mosaic H223R plus an insertion or deletion**	**3***	**0**	**1**	**4**	**yes[++]**	**4-9 months[+++]**
No DNA sequence		1	1	2	unknown	<10 days
No phenotype or unknown	5	9	3	17		
**Skeletal phenotype with the H223R mutation**	**5**	**2**	**1**	**8**		

* = two of three mosaic males (#8 and #9) were used for matings that yielded F1 pups

Suppl. Table 2: Mating scheme using males mosaic for *H223R-PTH1R* and *PTH1R-del*, genotypes and sex distribution of F1 pups

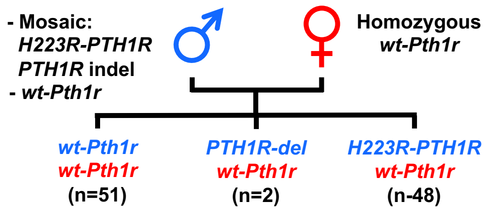

homozygous *wt-Pth1r* (males)	homozygous *wt-Pth1r* (females)	*PTH1R-del* *wt-Pth1r* (males)	*PTH1R-del* *wt-Pth1r* (females)	*H223R-PTH1R* *wt-Pth1r* (males)	*H223R-PTH1R* *wt-Pth1r* (females)
30	21	2	0	30	18

	H223R mutant (H223R-PTH1R/wt-Pth1r) Males+females	Wild-type (wt-Pth1r/wt-Pth1r) Males+females	H223R mutant (H223R-PTH1R/wt-Pth1r) Males alone	Wild-type (wt-Pth1r/wt-Pth1r) Males alone	H223R mutant (H223R-PTH1R/wt-Pth1r) Females alone	Wild-type (wt-Pth1r/wt-Pth1r) Females alone
**Calcium (mmol/L)**						
mean	2.71	3.13	2.64	3.26	2.88	2.75
SD	0.96	1.40	0.88	1.52	1.26	1.04
SEM	0.22	0.32	0.23	0.41	0.56	0.47
Median	2.73	2.77	2.60	3.04	2.99	2.77
n	19	19	14	14	5	5
p-value	0.5		0.3		0.6	
**Phosphate (mmol/L)**						
mean	2.84	3.12	3.10	2.99	2.38	3.51
SD	0.77	1.06	0.81	1.06	0.41	1.04
SEM	0.21	0.23	0.27	0.27	0.18	0.46
Median	2.74	3.01	2.77	3.13	2.40	3.01
n	14	21	9	16	5	5
p-value	0.4		0.4		0.7	
**PTH (pmol/L)**						
mean	5.45	7.99	3.63	7.79	7.26	8.54
SD	5.5	3.5	2.9	3.8	7.0	2.7
SEM	1.58	0.80	1.18	1.02	2.87	1.21
Median	2.62	7.29	2.56	6.81	3.36	8.12
n	12	19	6	14	6	5
p-value	0.01		0.001		0.4	
**CTX-1 (ng/ml)**						
mean	70.12	38.20	82.73	42.04	39.51	26.65
SD	79.2	32.5	88.5	26.3	39.7	47.9
SEM	16.16	6.63	21.48	6.20	14.99	19.54
Median	32.15	29.35	37.70	36.55	20.75	8.70
n	24	24	17	18	7	6
p-value	0.06		0.06		0.1	
**P1NP (ng/ml)**						
mean	13.39	31.44	11.50	36.16	17.71	17.27
SD	23.4	28.3	13.5	25.7	39.1	33.5
SEM	4.89	5.79	3.37	6.06	14.77	13.69
Median	2.81	24.17	6.91	31.55	1.94	3.94
n	23	24	16	18	7	6
p-value	0.003		0.001		0.2	
**CTX-2 (pg/ml)**						
mean	68.40	28.60	83.24	32.28	16.46	13.90
SD	64.4	18.5	65.9	18.2	6.6	12.9
SEM	15.18	4.77	17.62	5.25	3.29	7.44
Median	54.27	26.36	60.26	26.45	15.00	10.20
n	18	15	14	12	4	3
p-value	0.03		0.006		0.6	
